# Various meteorological conditions exhibit both immediate and delayed influences on the risk of stroke events: The HEWS–stroke study

**DOI:** 10.1371/journal.pone.0178223

**Published:** 2017-06-02

**Authors:** Tomoya Mukai, Naohisa Hosomi, Miwako Tsunematsu, Yoshimasa Sueda, Yutaka Shimoe, Tomohiko Ohshita, Tsuyoshi Torii, Shiro Aoki, Tomohisa Nezu, Hirofumi Maruyama, Masayuki Kakehashi, Masayasu Matsumoto, on behalf of the Hiroshima ‘Emergency and Weather’ Study-stroke collaborators

**Affiliations:** 1 Department of Clinical Neuroscience and Therapeutics, Hiroshima University Graduate School of Biomedical and Health Sciences, Hiroshima, Japan; 2 Department of Health Informatics, Hiroshima University Graduate School of Biomedical and Health Sciences, Hiroshima, Japan; 3 Department of Neurology, National Hospital Organization Kure Medical Center, Kure, Japan; 4 Department of Neurology, Brain Attack Center Ota Memorial Hospital, Fukuyama, Japan; 5 Department of Neurology, Suiseikai Kajikawa Hospital, Hiroshima, Japan; JAPAN

## Abstract

We hypothesized that meteorological conditions on the onset day and conditions on the former days may play important roles in the modulation of physical conditions. Associations of meteorological factors and their changes in former days with stroke onset are of interest. We conducted a multicenter retrospective study to evaluate the frequency of stroke events and their interaction with meteorological conditions and their daily changes. Acute stroke patients (n = 3935, 73.5±12.4 years, 1610 females) who were admitted to 7 stroke hospitals in three restricted areas were enrolled in this study. Poisson regression models involving time-lag variables was used to compare daily rates of stroke events with mean thermo-hydrological index (THI), atmospheric pressure, and their daily changes. We divided onset days into quintiles based on the THI, atmospheric pressure, and their daily changes for the last 7 days. The frequencies of ischemic stroke significantly increased when THI varied either cooler or warmer from a previous day (extremely cooler, risk ratio (RR) 1.19, 95% confidence interval (CI) 1.05 to 1.34; extremely warmer, RR 1.16, 95% CI 1.03 to 1.31; r^2^ = 0.001 for the best regression, p = 0.001). Intracerebral hemorrhage frequencies significantly decreased on high-THI days (extremely high, RR 0.72, 95% CI 0.54 to 0.95; r^2^ = 0.013 for the best regression, p<0.001) and increased in high atmospheric pressure days (high, RR 1.31, 95% CI 1.04 to 1.65; r^2^ = 0.009 for the best regression, p<0.001). Additionally, even after adjusting for the THI on the onset day and its changes for the other days, intracerebral hemorrhage increased when THI got extremely cooler in 4 days prior (RR 1.33, 95% CI 1.03 to 1.71, r^2^ = 0.006 for the best regression, p<0.001). Various meteorological conditions may exhibit influences on stroke onset. And, when temperature cooled, there may be a possibility to show delayed influence on the frequency of intracerebral hemorrhage 4 days later.

## Introduction

Numerous epidemiological studies have examined the relationships between cerebrovascular disease and meteorological conditions [1–3]. Seasonal variation significantly influences cerebrovascular diseases, which exhibit a high incidence in winter and a low incidence in summer [4–6]. A study from Japan found that intracerebral hemorrhage was more frequent on cold days [7]. Few articles have evaluated the relationship between the frequency of stroke onset and atmospheric pressure [2, 8]. Decreased atmospheric pressure increases the risk of non-lacunar stroke, and increased atmospheric pressure increases the risk of the onset of intracerebral hemorrhage [2].

Complicated mechanisms regulate pathophysiological homeostasis. Physical responses to environmental changes may also be delayed. Several studies have reported a delayed relationship between temperature changes and an increased risk of cerebrovascular diseases [9–11]. Increased risk of ischemic stroke was reported 2 to 5 days after cold exposure [9, 12]. We hypothesized that meteorological conditions on the onset day and conditions on the former days may play important roles in the modulation of physical conditions. To our knowledge, our previous small sized study is the only study that has evaluated the influence of daily changes in meteorological conditions on stroke onset [13]. The results of this study suggest a higher risk for ischemic stroke events with low ambient temperature and elevated temperature on a previous day. However, an interaction between ambient temperature or atmospheric pressure and their changes was not evaluated because of the small sample size of this study.

Therefore, associations of meteorological factors and their changes in a former day with stroke onset are of interest. We conducted a multicenter retrospective study to evaluate the frequency of stroke events and their interaction with meteorological conditions and their daily changes.

## Materials and methods

### Patients

The protocol was approved by the Institutional Review Board in Hiroshima University. Then, the local ethical committee in each participating institute approved the protocol. All clinical investigation was conducted according to the principles expressed in the Declaration of Helsinki. Because the data were analyzed anonymously, no informed consent was given. We enrolled consecutive acute stroke patients who were admitted to seven emergency hospitals in the Hiroshima prefecture, which included the cities of Hiroshima (four hospitals), Kure (two hospitals), and Fukuyama (one hospital), from January 2012 to December 2013. These three cities contained the official meteorological observatories that were used as the source of meteorological data. The onset date and subtype of acute strokes were recorded for each patient. Ischemic stroke subtypes were classified using the Trial of Org 10172 in Acute Stroke Treatment (TOAST) criteria [14]. The etiology of the qualifying ischemic event was classified as cardioembolic infarction (high and medium risk cardiac-source for cerebral emboli), atherothrombotic infarction (occlusion or >50% stenosis of an appropriate large extracranial or intracranial artery or occlusion of appropriate stem, division or branch artery), lacunar infarction, and the others.

Hemorrhagic infarction and trauma-induced hemorrhage were excluded from intracerebral hemorrhage. The primary diseases of non-hypertensive intracerebral hemorrhage included tumor, arteriovenous malformation, moyamoya disease, and cerebral amyloid angiopathy. Stroke subtype diagnoses were determined before discharge using echocardiography, brain computed tomography, magnetic resonance imaging (MRI), magnetic resonance angiography and/or carotid ultrasonography. Hypertension was defined as the use of anti-hypertensive medications prior to admission or a confirmed blood pressure of ≥140/90 mmHg at rest 2 weeks after stroke onset. Diabetes mellitus was defined as glycated hemoglobin levels of ≥6.5%, fasting blood glucose levels of ≥126 mg/dl, or the use of anti-diabetic medication. Dyslipidemia was defined as total cholesterol levels of ≥220 mg/dl, low-density lipoprotein cholesterol levels of ≥140 mg/dl, high-density lipoprotein cholesterol levels of <40 mg/dl, triglyceride levels of ≥150 mg/dl, or the use of anti-hyperlipidemia medication. Expert neurologists assigned the diagnosis in all cases.

### Study area

Japan is located in a temperate climate zone with four distinct seasons: spring, summer, autumn, and winter. The Hiroshima prefecture is located in the western part of Japan (North latitude 34–35 degrees and East longitude 132–133 degrees) in a Cfa zone (warm temperature, moist, hot summer), based on Köppen-Geiger climate classification [15].

### Meteorological data

Meteorological data in this study included daily mean ambient temperature (Ta, °C), daily mean atmospheric pressure (hPa), and daily mean relative humidity (RH, %) of the 24-hr calendar day period (0:00AM-11:59PM) on the onset day and the last 7 days, which were obtained from the local meteorological observatories (Japan Meteorological Agency, Ministry of Land, Infrastructure, Transport and Tourism). The thermo-hydrological index (THI, °C) was calculated using the formula

THI=Ta−0.55*(1−0.01*RH)*(Ta−14.5),

as reported previously [13]. This index is an established appropriate measure for the evaluation of the effect of air temperature on health outcomes because it takes into account mean air temperature after controlling for the effect of relative humidity. The distance from each hospital to the local meteorological observatory ranged from 0.2 km to 12.8 km (average distance = 5.3 km). All meteorological data were obtained from the website of each local meteorological observatory.

The daily rates of acute stroke events for each city were compared to the mean THI (T_0_), mean atmospheric pressure (P_0_) of the onset day, and their daily changes for the last 7 days. Daily THI changes were calculated, e.g. from a previous day to the onset day (T_0_–T_1_), from 2 days prior to the onset day to the previous day (T_1_–T_2_), and so on. Daily atmospheric pressure changes were calculated, e.g. from a previous day to the onset day (P_0_–P_1_), from 2 days prior to the onset day to the previous day (P_1_–P_2_), and so on.

We divided the day of interest into quintiles based on the mean THI (T_0_; extremely low (EL) temperature, ≤7.9°C; low (L) temperature, 8.0–12.7°C; intermediate (I) temperature, 12.8–18.6°C; high (H) temperature, 18.7–23.7°C, and extremely high (EH) temperature, ≥23.8°C) and the mean atmospheric pressure (P_0_; EL pressure, ≤1006.6 hPa; L pressure, 1006.7–1010.7 hPa; I pressure, 1010.8–1014.6 hPa; H pressure, 1014.7–1019.1 hPa; and EH pressure, ≥1019.2 hPa). The analyzed days were divided into quintiles based on daily changes in the mean THI for the last 7 days (T_0_-T_1_ and so on; extremely cooler (EC), ≤-0.99°C; cooler (C), -0.98–-0.21°C; unchanged (U), -0.20–0.31°C; warmer (W), 0.32–0.98°C, and extremely warmer (EW), ≥0.99°C). The examined days were also divided into quintiles based on daily changes in the atmospheric pressure for the last 7 days (P_0_-P_1_ and so on; extremely decreased (ED), ≤-2.8 hPa; decreased (D), -2.79–-0.70 hPa; unchanged (U), -0.69–1.00 hPa; increased (In), 1.01–3.20 hPa; and extremely increased (EIn), ≥3.21 hPa).

### Statistical analysis

Data are expressed as means±standard deviation (SD) for continuous variables and as frequencies and percentages for discrete variables. We first investigated basic trends between incidence rates and meteorological variables (THI and atmospheric pressure) on the onset day using linear regression analyses.

We then used a multivariable Poisson regression model involving time-lag variables instead of a common multiple regression model in analyses of the incidence of stroke because the response variables were count data or non-negative integers. The risk ratio and the 95% confidence interval (CI) of stroke in accordance with temperature and pressure were estimated, controlling for the location of the hospitals using the Poisson regression model.

The response variables were the daily incidences of each stroke. Explanatory variables, other than the effect of location, were segmented into five quintiles of observation days. The effects of temperature and pressure on the main effects and interactions were examined in the most detailed model. We also used the daily changes of THI or atmospheric pressure for the last 7 days as time-lag variables to examine the effects of increases/decreases in THI or atmospheric pressure in each day. These variables were also segmented into quintiles of observation days as mentioned above. The model equation is described as follows:

$$\text{ln}\lambda =a+b_{location}+b_{temperature}+b_{difference1}+\cdot \cdot \cdot ,$$

where λ denotes Poisson parameter (the mean of Poisson distribution) and ln represents natural logarithm. Coefficients *b*’s are the effects of meteorological variables and *a* is an intercept. Additionally, we have made a best regression analysis in raw data where we found a statistical significance with a multivariable Poisson regression model.

Statistical analysis was performed using the SPSS software package (IBM SPSS Statistics for Windows, version 22.0, IBM Corp., Armonk, NY, USA). A two-tailed p of <0.05 was regarded as statistically significant.

## Results

### Patient demographics

Table 1 presents the baseline characteristics of the study population. A total of 3935 patients (1610 females and 2325 males) with stroke onset were enrolled during the 2-year observation period of this study. The mean age of the study participants was 73.5±12.4 years (median 75 years; range 11–101 years). The subjects consisted of 3197 ischemic stroke and 738 intracerebral hemorrhage patients. Ischemic stroke was classified as cardioembolic stroke (806/3935, 20.5%), atherothrombotic infarction (959/3935, 24.4%), and lacunar infarction (672/3935, 17.1%). Intracerebral hemorrhage was classified as hypertensive intracerebral hemorrhage (626/3935, 15.9%) and non-hypertensive intracerebral hemorrhage (112/3935, 2.8%).

**Table 1:** Baseline characteristics.

Factors	n = 3935
**Age**	73.5±12.4
**Male sex, n (%)**	2325 (59.1)
**Stroke subtype, n (%)**	
Ischemic stroke	3197 (81.2)
Cardioembolic stroke	806 (20.5)
Atherothrombotic infarction	959 (24.4)
Lacunar infarction	672 (17.1)
Others	760 (19.3)
Intracerebral hemorrhage	738 (18.8)
Hypertensive intracerebral hemorrhage	626 (15.9)
Non-hypertensive intracerebral hemorrhage	112 (2.8)
**Location**	
Hiroshima, n (%)	1640 (41.7)
Kure, n (%)	613 (15.6)
Fukuyama, n (%)	1682 (42.7)
**Risk factors**	
Hypertension, n (%)	2952 (75.0)
Diabetes mellitus, n (%)	1120 (28.5)
Dyslipidemia, n (%)	1710 (43.5)
Atrial fibrillation, n (%)	782 (19.9)

The annual mean THI of the three cities was 16.0±7.3°C. The monthly mean THI ranged from 6.5±1.6°C in January to 26.6±1.1°C in August. The annual mean atmospheric pressure in the study area was 1012.6±7.1 hPa. The monthly mean atmospheric pressure ranged from 1005.9±5.4 hPa in June to 1019.6±5.4 hPa in January. The annual mean THI and atmospheric pressure were not significantly different among the three cities.

### Association of the stroke event rate with THI and atmospheric pressure on the onset day

**Fig 1:**
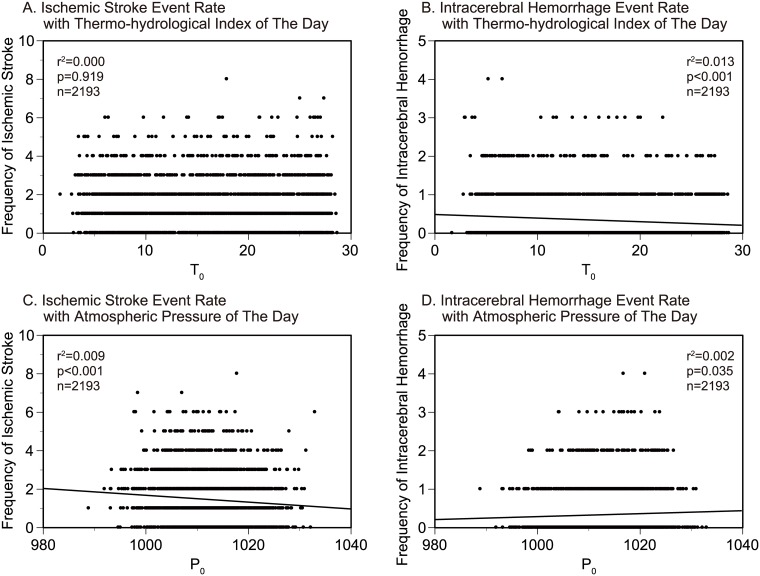
Association of the stroke event rates with mean thermo-hydrological index and mean atmospheric pressure on the onset day. Event rate of ischemic stroke (A) and intracerebral hemorrhage (B) with mean THI of the onset day (T_0_), and event rate of ischemic stroke (C) and intracerebral hemorrhage (D) with mean atmospheric pressure (P_0_) were presented.

The frequency of ischemic stroke showed no apparent trend with T_0_ (r^2^ = 0.000, p = 0.919; Fig 1A). On the other hand, the frequency of intracerebral hemorrhage decreased significantly in warm days (r^2^ = 0.013, p<0.001; Fig 1B).

The frequency of ischemic stroke decreased and intracerebral hemorrhage increased in high atmospheric pressure days (r^2^ = 0.009, p<0.001; r^2^ = 0.002, p = 0.035; Fig 1C and 1D).

### Association between the frequency of stroke events and daily changes in THI

**Fig 2:**
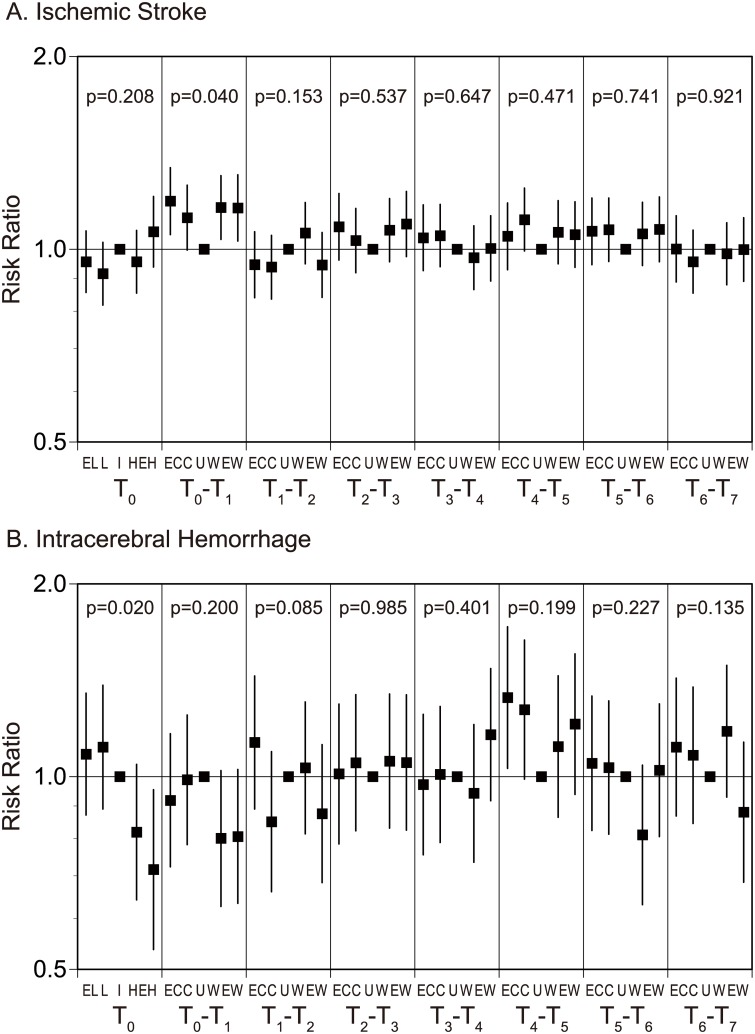
Risk ratio of ischemic stroke (A) and intracerebral hemorrhage (B) with THI of the onset day and its daily changes for the last 7 days. EL, extremely low temperature, ≤7.9°C; L, low temperature, 8.0–12.7°C; I, intermediate temperature, 12.8–18.6°C; H, high temperature, 18.7–23.7°C; EH, extremely high temperature, ≥23.8°C; EC, extremely cooler, ≤-0.99°C; C, cooler, -0.98–-0.21°C; U, unchanged, -0.20–0.31°C; W, warmer, 0.32–0.98°C; and EW, extremely warmer, ≥0.99°C.

**Fig 3:**
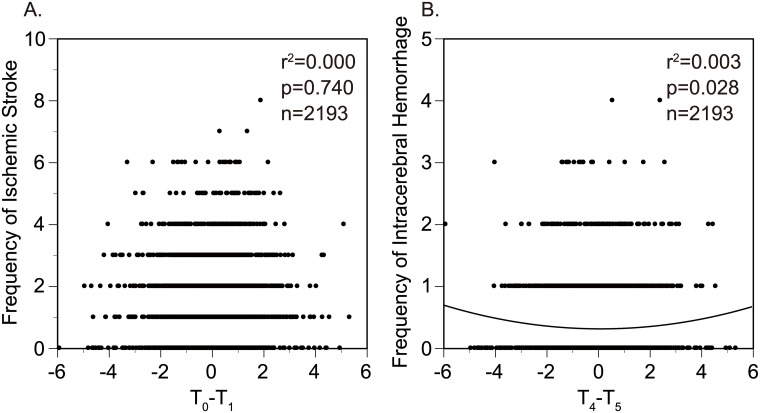
Scatter diagrams of ischemic stroke rates with T_0_-T_1_ (A) and intracerebral hemorrhage with T_0_-T_1_ (B).

To define the association of daily THI changes on the stroke onset, we evaluated the risk ratio of stroke onset with THI of the onset day (T_0_) in combination with the daily THI changes for the last 7 days using the multivariable Poisson regression models involving time-lag variables. Ischemic stroke events were associated with THI and its changes (p<0.001; Fig 2A). The frequency of ischemic stroke increased when THI varied either cooler or warmer from a previous day to the onset day (T_0_–T_1_ EC, RR 1.19, 95% CI 1.05 to 1.34; W, RR 1.16, 95% CI 1.04 to 1.30; and EW, RR 1.16, 95% CI 1.03 to 1.31). And, it increased when THI got cooler in 4 days prior to the onset day (T_4_–T_5_ C, RR 1.11, 95% CI 0.99 to 1.25), although it was not significant. When evaluating association of the frequency of ischemic stroke with T_0_-T_1_ in a scatter diagram, there was no significant associations in it (r^2^ = 0.000 for the best regression, p = 0.740, Fig 3A).

Intracerebral hemorrhage events were associated with THI and its changes (p<0.001, Fig 2B). Intracerebral hemorrhage decreased on extremely-high temperature days (T_0_ EH, RR 0.72, 95% CI 0.54 to 0.95). And, it increased when THI got extremely cooler in 4 days prior to the onset day (T_4_–T_5_ EC, RR 1.33, 95% CI 1.03 to 1.71). When evaluating association of the frequency of intracerebral hemorrhage with T_4_-T_5_ in a scatter diagram, there was also a significant association in it (r^2^ = 0.003 for the best regression, p = 0.028, Fig 3B).

### Association between the frequency of stroke events and daily changes in atmospheric pressure

**Fig 4:**
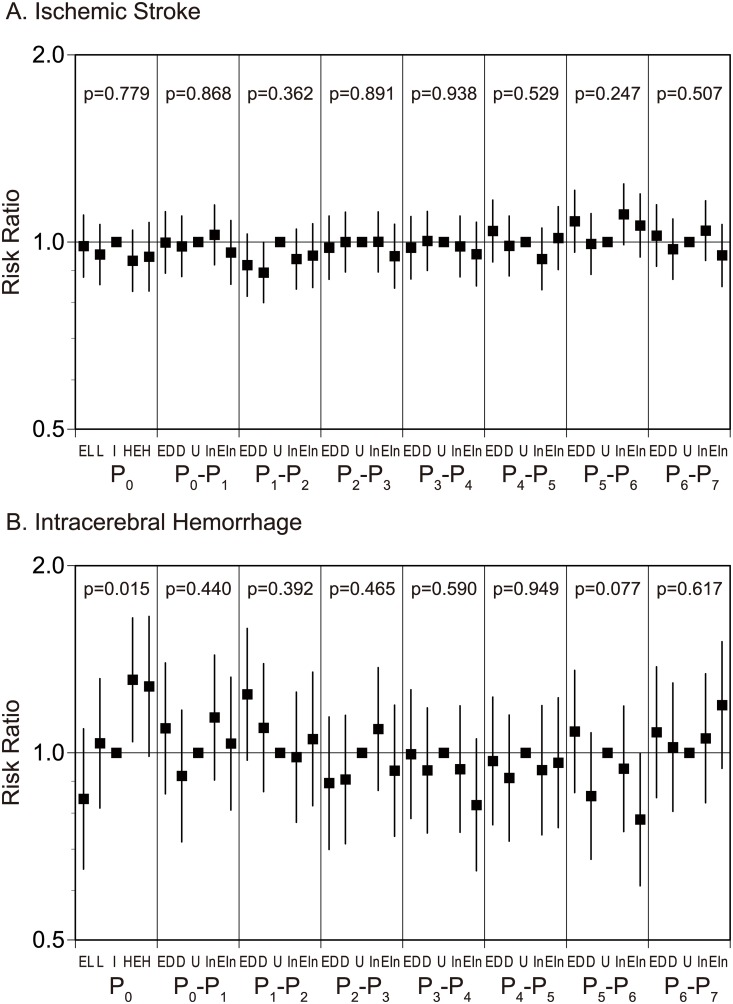
Risk ratio of ischemic stroke (A) and intracerebral hemorrhage (B) with atmospheric pressure of the onset day and its daily changes for the last 7 days. EL, extremely low pressure, ≤1006.6 hPa; L, low pressure, 1006.7–1010.7 hPa; I, intermediate pressure, 1010.8–1014.6 hPa; H, high pressure, 1014.7–1019.1 hPa; EH, extremely high pressure, ≥1019.2 hPa; ED, extremely decreased, ≤-2.8 hPa; D, decreased, -2.79–-0.70 hPa; U, unchanged, -0.69–1.00 hPa; In, increased, 1.01–3.20 hPa; and EIn, extremely increased, ≥3.21 hPa).

To define the association of the daily atmospheric pressure changes on the stroke onset, we evaluated the risk ratio of stroke onset with atmospheric pressure of the onset day (P_0_) in combination with its daily changes for the last 7 days using the multivariable Poisson regression models involving time-lag variables. Ischemic stroke events were associated with atmospheric pressure and its changes (p<0.001, Fig 4A). However, there was no factor, which significantly associate with ischemic stroke onset among the atmospheric pressure of the onset day (P_0_) and its daily changes for the 7 days. Intracerebral hemorrhage events were associated with atmospheric pressure and its changes (p<0.001, Fig 4B). Intracerebral hemorrhage increased in high atmospheric pressure days (P_0_ H, RR 1.31, 95% CI 1.04 to 1.65). No association was observed from its daily changes.

## Discussion

The present study detected relevant relationships between the frequency of stroke and meteorological conditions. The frequency of ischemic stroke increased when THI varied either cooler or warmer from a previous day. And, the frequency of intracerebral hemorrhage decreased in an extremely-high temperature days, and increased in high-pressure days and when THI got extremely-cooler in 4 days prior to the onset day.

Previous reports have suggested that the incidence of intracerebral hemorrhage exhibits seasonal variations, with higher rates in winter and lower rates in summer [7]. Certain studies have indicated that intracerebral hemorrhage is more relevant on days with low ambient temperatures [16]. The findings of these prior investigations are consistent with our results. Our results suggest that a low onset rate of intracerebral hemorrhage is observed in warm days. It has been reported that intracranial hemorrhage is strongly associated with the high blood pressure [17]. And, naturally, blood pressure decreases in summer [18]. Therefore, this blood pressure reduction may account for a low onset rate of intracerebral hemorrhage in warm days.

We hypothesized that ischemic stroke frequently occurred on hot or cold days. However, our results demonstrate no association of ischemic stroke with THI of the onset day. On the other hand, we observe a high risk ratio of ischemic stroke with changes of THI from a previous day to the onset day either cooler or warmer. Our results indicate that ambient temperature variation influences the frequency of ischemic stroke events. The human body maintains homeostasis during numerous environmental changes, e.g. ambient temperature, atmospheric pressure, and sunlight. Serial changes in physical conditions affect health conditions [18, 19], such as blood pressure and blood glucose fluctuations, which adversely affect stroke patient outcomes [20, 21]. Our results consistently demonstrate that the frequencies of ischemic stroke and intracerebral hemorrhage exhibit high risk ratio when temperature become cooler in 4 days prior to the onset. This result may reflect the carryover effects of changes in ambient temperature over days prior to onset. This environmental variation in daily ambient temperature may cause a malfunction of the body’s adjustment to changes [18, 19]. Hypertensive states caused by peripheral vessel contraction and sympathetic nerve dominancy may play a partial role in this malfunction.

We also detect certain associations of intracerebral hemorrhage with atmospheric pressure. It is difficult to define whether ambient temperature or atmospheric pressure primarily associates with intracerebral hemorrhage onset because these factors are linearly associated to each other (S1 Fig). Ambient temperature and atmospheric pressure exhibit reciprocal interactions in seasonal changes. A high ambient temperature with low atmospheric pressure is observed in summer, and a low ambient temperature with high atmospheric pressure is observed in winter. The meteorological relationship between atmospheric pressure and ambient temperature suggests that these factors have opposite impacts on intracerebral hemorrhage onset.

There are several limitations to our study that should be mentioned. First, this report is a retrospective study of restricted areas in the Hiroshima prefecture in Japan. However, this study and prior epidemiological studies [22] are similar with respect to patient demographics, such as stroke subtype and age; as a result, our findings may be applicable for other cities. Second, the examined cities differ in economic conditions, population, and meteorological conditions. Although we have adjusted by location in analyses of the influence of meteorological conditions on stroke incidence, differences among locations may still influence the ability of meteorological conditions to predict stroke incidence. However, the examined cities are extremely close geographically and experience similar meteorological conditions. Third, as we have evaluated associations of the mean ambient temperature and atmospheric pressure on a stroke occurrence in the same day, there may be a potent number of subjects who did not suffer to these meteorological conditions depend on the onset timing. However, in case of ambient temperature (e.g. THI), it was varied 0.0±1.4°C from the previous day (data not shown). The latest 24 hour ambient temperature was quite similar with the onset day. Therefore, this limitation may show a quite limited influence. Fourth, given limitations of statistical analysis, we could not include classical risk factors for adjustment, because those personal factors could not fit to the daily factors. Therefore, considerable arguments on the proportional bias of subjects with those risk factors in the high frequency days. Therefore, we have additionally evaluated the proportion of subjects with elderly, male, and hypertension where we found a statistically significance in a Poisson regression model (S1 Tables). And, we found the subjects with those risk factors did not biased in those conditions. Fifth, this study is based on patients admitted to hospitals. We cannot exclude the possibility that patients who experienced the onset of a mild stroke may not have visited a hospital. This limitation may also exist in population-based studies. However, in Japan, when patients with mild strokes visit a hospital for diagnosis in the acute phase, they are essentially admitted and allowed to receive treatment. Therefore, the possibility that patients with mild strokes were excluded from this study may be of limited concern. Sixth, we could not include individual information regarding true environmental status (indoor and outdoor), socioeconomic background, or dietary habits at onset. Seventh, this study was conducted in Japan, and almost all of the enrolled patients were Japanese. Asians suffer more hemorrhagic events than Caucasians [22]. There are known ethnic differences in the tolerance toward environmental changes. For example, Hispanics are more likely to be affected by temperature [23]. Further investigations should be performed to determine ethnic differences.

## Conclusions

Associations between meteorological conditions and their changes with stroke onset were evaluated. Our results suggest that higher temperature on the onset day is associated with the low frequencies of intracerebral hemorrhage events. Varied meteorological conditions may exhibit some associations on ischemic stroke onset. And, when temperature get cooled, it may show delayed influence on the frequency of intracerebral hemorrhage 4 days later. The meteorological factors, ambient temperature and atmospheric pressure, have different impacts on intracerebral hemorrhage onset in contrast to ischemic stroke.

## Supporting information

S1 Fig Association of atmospheric pressure and thermo-hydrological index. There was a linear association between atmospheric pressure and thermo-hydrological index (THI).
(EPS)

S1 Tables Proportion of elderly, male, and hypertension in T_0_-T_1_ for ischemic stroke subjects and T_0_, T_4_-T_5_, and P_0_ for intracerebral hemorrhage subjects. (PDF)
