# Supplementary material for: Various meteorological conditions exhibit both immediate and delayed influences on the risk of stroke events: The HEWS–stroke study
Source: PLoS One. 2017 Jun 2;12(6):e0178223. doi: 10.1371/journal.pone.0178223 (PMC5456042; doi:10.1371/journal.pone.0178223)
Supplement: S1 Tables — (PDF) [file pone.0178223.s002.pdf]

Supplemental Tables. Proportion of elderly, male, and hypertension.

For ischemic stroke subjects,

	T_0-T_1					
	EC<br>n=672	C<br>n=626	U<br>n=570	W<br>n=664	EW<br>n=665	χ^2 test
Elderly, n (%)	360<br>(53.6)	329<br>(52.6)	308<br>(54.0)	348<br>(52.4)	361<br>(54.3)	p=0.95
Male, n (%)	398<br>(59.2)	369<br>(58.9)	348<br>(61.1)	420<br>(63.3)	394<br>(59.2)	p=0.45
Hypertension, n (%)	492<br>(73.2)	437<br>(69.8)	394<br>(69.1)	456<br>(68.7)	494<br>(74.3)	p=0.08

EC, extremely cooler; C, cooler; U, unchanged; W, warmer; EW, extremely warmer.

For intracerebral hemorrhage subjects,

	T_0					
	EL<br>n=182	L<br>n=167	I<br>n=155	H<br>n=125	EH<br>n=109	χ^2 test
Elderly, n (%)	74 (40.7)	70 (41.9)	74 (47.7)	54 (43.2)	38 (34.9)	p=0.33
Male, n (%)	106 (58.2)	79 (47.3)	77 (49.7)	66 (52.8)	68 (62.4)	p=0.07
Hypertension, n (%)	169 (92.9)	154 (92.2)	140 (90.3)	117 (93.6)	99 (90.8)	p=0.84

EL, extremely low; L, low; I, intermediate; H, high; EH, extremely high.

	T_4-T_5					
	EC<br>n=175	C<br>n=149	U<br>n=111	W<br>n=138	EW<br>n=165	χ^2 test
Elderly, n (%)	70 (40.0)	55 (36.9)	50 (45.0)	61 (44.2)	74 (44.8)	p=0.54
Male, n (%)	83 (47.4)	87 (58.4)	64 (57.7)	78 (56.5)	84 (50.9)	p=0.22
Hypertension, n (%)	158<br>(90.3)	136<br>(91.3)	102<br>(91.9)	126<br>(91.3)	157<br>(95.2)	p=0.54

EC, extremely cooler; C, cooler; U, unchanged; W, warmer; EW, extremely warmer.

	P_0					
	EL n=132	L n=144	I n=135	H n=175	EH n=152	χ^2 test
Elderly, n (%)	58 (43.9)	55 (38.2)	54 (40.0)	69 (39.4)	74 (48.7)	p=0.34
Male, n (%)	72 (54.5)	82 (56.9)	75 (55.6)	92 (52.6)	75 (49.3)	p=0.72
Hypertension, n (%)	117 (85.6)	135 (93.7)	124 (91.9)	161 (92.0)	142 (93.4)	p=0.55

EL, extremely low; L, low; I, intermediate; H, high; EH, extremely high.
